# The Natural Course of Bosch‐Boonstra‐Schaaf Optic Atrophy Syndrome

**DOI:** 10.1111/cge.14731

**Published:** 2025-02-19

**Authors:** Ilia Valentin, Pilar Caro, Christine Fischer, Heiko Brennenstuhl, Christian P. Schaaf

**Affiliations:** ^1^ Institute of Human Genetics University Heidelberg Heidelberg Germany

**Keywords:** BBSOAS, developmental delay, intellectual disability, NR2F1, visual impairment

## Abstract

(Likely) pathogenic variants in *NR2F1* are associated with Bosch‐Boonstra‐Schaaf optic atrophy syndrome (BBSOAS, OMIM #615722), a rare neurodevelopmental disorder. Patients present with a variety of symptoms, including intellectual disability, developmental delay, visual impairment, muscular hypotonia, seizures, and/or autistic features. Since it was first described in 2014, the phenotype has steadily expanded. However, there is limited information regarding the natural course of the disorder. Here, we present data on genetic variants and phenotype development of 47 individuals who responded to our questionnaire. A questionnaire was developed to assess the phenotype more comprehensively and to better understand the course of symptoms. In addition, families sent medical documents and photographs. To investigate the genotype‐phenotype correlation in our cohort, we compared clinical features of two genotypically distinct groups (variants in the DNA‐binding domain (DBD, *n* = 17) versus variants elsewhere in the gene (*n* = 30)). We observed a range of common symptoms including developmental delay, muscular hypotonia, optic atrophy, nystagmus, strabismus, autistic features, and thin corpus callosum on brain MRI. Overall, more improvement than worsening was reported. Individuals with variants in the DBD showed a higher prevalence of severe clinical features, such as infantile spasms (46.7% vs. 3.8%, *p* = 0.002) or nonverbality (50% vs. 3.4%, *p* = 0.0004), age at diagnosis was statistically significantly different between the two genotypic groups (mean 4.7 years vs. 8.9 years, *p* = 0.048). Our study confirms characteristic clinical features of BBSOAS. Variants in the DBD are associated with a more severe clinical phenotype. We found no evidence that the disease progresses; rather, several symptoms are reported to improve over time. However, prospective longitudinal studies are needed to further investigate disease progression.

## Graphical abstract

Here, we present data on genetic variants and phenotype development of 47 individuals with Bosch‐Boonstra‐Schaaf optic atrophy syndrome, a rare neurodevelopmental disorder, highlighting the fact that the disease does not appear to be fundamentally progressive. Moreover, variants in the DNA‐binding domain are associated with a more severe clinical phenotype.

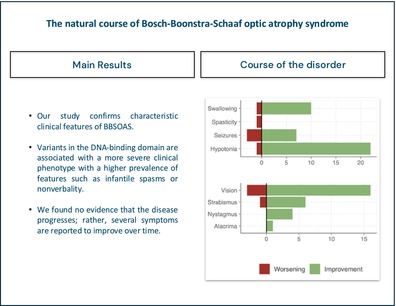

## 1 Introduction

Bosch‐Boonstra‐Schaaf optic atrophy syndrome (BBSOAS, OMIM #615722) is a neurodevelopmental disorder caused by pathogenic variants in *NR2F1* [1]. In 2014, Bosch et al. described the first six individuals with heterozygous *NR2F1* missense variants or gene deletions. All showed optic nerve abnormalities and intellectual impairment [2]. In the following years, the clinical picture of BBSOAS widened. Further ophthalmological features such as cerebral visual impairment (CVI), strabismus, nystagmus, alacrima, and/or refractive errors have become known to be part of the clinical phenotype [1, 3]. Moreover, muscular hypotonia, oromotor dysfunctions, seizures including infantile spasms, behavioral abnormalities, and autism spectrum disorder are commonly associated with BBSOAS [1, 4]. When present, facial dysmorphism seems to be nonspecific [4, 5].

*NR2F1*, also known as *COUP‐TFI*, encodes a highly conserved orphan nuclear receptor protein which acts as a transcriptional regulator [6]. Previous studies showed that Nr2f1 plays a fundamental role in the development of the murine brain [7, 8] and components of the visual system [9]. Analogous to other nuclear receptors, NR2F1 comprises two highly conserved domains: a DNA‐binding domain (DBD) and a ligand‐binding domain (LBD) [6]. Disease‐causing missense variants are preferentially found in these two domains [4, 10]. Variants in the DBD, comprising two Zinc‐finger motifs [11], have been associated with more severe clinical phenotypes [1], with an underlying dominant negative effect as a possible explanation [4].

So far, 112 sequence variants in *NR2F1* have been published [5], the phenotype of BBSOAS has been described in 92 individuals [10] and literature is constantly growing. We developed a questionnaire to assess the phenotype more comprehensively. Additionally, our study provides insights into the course of the symptoms. Here we present data of 47 individuals who answered our questionnaire.

## 2 Subjects and Methods

To collect data about the phenotype and its course, we developed a structured questionnaire in two languages (English and German). Patients of the BBSOAS registry at University Hospital Heidelberg were contacted via email by the research coordinator to inform them about the study. Additionally, the research study was announced in the closed Facebook group for BBSOAS Families and shared among the NR2F1 association. We received 48 completed questionnaires. One subject was excluded as the individual's *NR2F1* variant was evaluated as likely benign, and the final number of subjects enrolled in the study was 47.

Inclusion criteria was a previously identified variant in *NR2F1*. There were no exclusion criteria. The study was performed as part of the human research study H‐34578 (Understanding the Molecular Causes of Neuropsychiatric Disease), which was approved by the Baylor College of Medicine Institutional Review Board. Families were sent a consent form, a clinical questionnaire, and were asked to share medical information and photographs. Written informed consent for publication of their clinical details was obtained from the individual's parents or guardians.

To evaluate the course of disease, respondents were asked whether symptoms had changed over time (worsened/improved/no change). To investigate the genotype–phenotype correlation in our cohort, we compared the clinical data of individuals with variants in the DBD (*n* = 17) and those with variants elsewhere in the gene (non‐DBD, *n* = 30). We further divided the genotypes into three groups: variants in the DBD (*n* = 17), in the translation‐initiation codon (TIC) and whole gene deletions (WGD) (*n* = 12), as well as variants in the LBD and elsewhere in the gene (*n* = 18). The comparison between these three genotypic groups is shown in Table S2.

We assessed differences between DBD and non‐DBD patients for categorical variables using Chi‐square tests or Fisher's exact tests in the case of sparse data. For quantitative variables Student's t‐tests were applied. To analyze age‐at‐event variables, we included patients with and without the event as censored observations and used their age at last observation. We present Kaplan‐Meier estimates of cumulative distribution functions. For assessing the differences between genetic groups, we used Log‐rank tests. All p‐values are two‐sided, and *p* < 0.05 was considered significant (no adjustment for multiple comparisons). A comprehensive list of confidence intervals by genotypic groups is provided in Table S1. Analyses were performed using SPSS V27 (IBM Corp. Armonk, NY, USA) BIAS V 11.4 (epsilon‐Verlag GbR Hochheim, Darmstadt, Germany), and R V4.1.3 (R Core Team (2020)). R: A language and environment for statistical computing. R Foundation for Statistical Computing, Vienna, Austria. URL https://www.R‐project.org/.

## 3 Results

The entire study cohort includes 28 females and 19 males with a mean age of 10.6 years, ranging from 0.7 to 27.5 years (SD 6.9, age at the time of completing the questionnaire).

### 3.1 Genotypes

**FIGURE 1:**
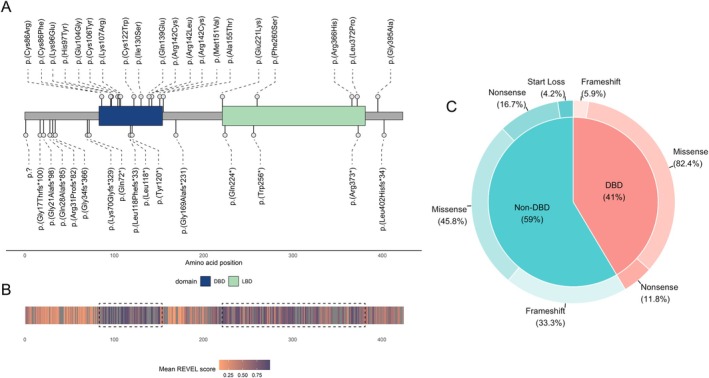
(A) Model of *NR2F1* gene showing locations of sequence variants of individuals enrolled in our study. Large deletions/duplications are not shown. Important domains are highlighted in blue (DBD) and green (LBD). (B) Heatmap of mean REVEL scores along the linearized NR2F1 protein. Functionally relevant domains are highlighted with dashed lines. (C) Frequency of variant types depending on the domain. Whole gene deletions are not included. The majority of variants in the DBD are missense variants, while outside the DBD missense, nonsense and frameshift variants are relatively balanced.

A total number of 47 individuals with a clinical phenotype of BBSOAS were enrolled in this study: 45 patients with molecularly confirmed BBSOAS (pathogenic/likely pathogenic variant in *NR2F1*) and two individuals with a variant of unknown significance. Additional genetic variants were found in 12 individuals. Most of the variants were determined to be de novo (60%), and the inheritance of the rest remained unknown. There were no individuals in this cohort whose variant was known to be inherited. Seventeen individuals showed variants in the DBD (36.2%), 7 had variants in the LBD (14.9%), 6 had variants in the TIC (12.8%), another 6 had WGD (12.8%) and 11 individuals had variants elsewhere in the gene (23.4%). An overview of *NR2F1* genotypes of the present study is shown in Table 1 and Figure 1.

**TABLE 1:** Overview of *NR2F1*‐genotypes of the present study.

Age (years)	Inheritance	Genomic position (hg38, NC_000005.10)	HGVSc (NM_005654.6)	HGVSp (NP_005645.1)	Variant Type	Protein domain	*In silico* prediction (REVEL, CADD)
10	De novo	g.93585224_93585230dup	c.201_207dup	p.(Lys70Glyfs*329)	Frameshift	EW	‐, −
4	De novo	g.93585474A>G	c.451A>G	p.(Met151Val)	Missense	DBD	0.965, 24.7
11	Unknown	Whole Gene Deletion	—	—	Whole Gene Deletion	WGD	—, —
10	Unknown	g.93593685T>C	c.1115T>C	p.(Leu372Pro)	Missense	LBD	0.953, 32
6	De novo	g.93585447C>T	c.424C>T	p.(Arg142Cys)	Missense	DBD	0.979, 32
9	De novo	g.93585025T>C	c.2T>C	p.(Met1Thr)	Missense	TIC	0.53, 22.5
17	De novo	g.93585025T>C	c.2T>C	p.(Met1Thr)	Missense	TIC	0.53, 22.5
10	De novo	g.93585448G>T	c.425G>T	p.(Arg142Leu)	Missense	DBD	0.976, 32
5	De novo	g.93585113_93585131del	c.90_108del	p.(Arg31Profs*82)	Frameshift	EW	‐, −
2	De novo	g.93593667G>A	c.1097G>A	p.(Arg366His)	Missense	LBD	0.907, 32
22	De novo	g.93593754G>C	c.1184G>C	p.(Gly395Ala)	Missense	EW	0.551, 29.6
3	De novo	Whole gene deletion	—	—	Whole Gene Deletion	WGD	—, —
16	De novo	g.93587944_93587953dup	c.491_500dup	p.(Gly169Alafs*231)	Frameshift	EW	—, —
22	De novo	g.93585071_93585077del	c.48_54del	p.(Gly17Thrfs*100)	Frameshift	EW	—, —
12	Unknown	g.93585447C>T	c.424C>T	p.(Arg142Cys)	Missense	DBD	0.979, 32
20	De novo	g.93585025T>C	c.2T>C	p.(Met1Thr)	Missense	TIC	0.53, 22.5
10	Not maternal	g.93593775_93593776del	c.1205_1206del	p.(Leu402Hisfs*34)	Frameshift	EW	—, —
13	De novo	g.93588114G>A	c.661G>A	p.(Glu221Lys)	Missense	LBD	0.823, 32
4	De novo	g.93585334A>G	c.311A>G	p.(Glu104Gly)	Missense	DBD	0.941, 32
12	Unknown	g.93585237C>T	c.214C>T	p.(Gln72*)	Nonsense	EW	—, 36
6	De novo	g.93585279T>C	c.256T>C	p.(Cys86Arg)	Missense	DBD	0.95, 32
14	De novo	g.93588182_93588183delinsCT	c.729_730delinsCT	p.Gln224*	Nonsense	LBD	—, —
10	De novo	g.93585438C>G	c.415C>G	p.(Gln139Glu)	Missense	DBD	0.903, 26
17	De novo	g.93585025T>C	c.2T>C	p.(Met1Thr)	Missense	TIC	0.53, 22.5
11	Unknown	g.93585312C>T	c.289C>T	p.(His97Tyr)	Missense	DBD	0.961, 27.1
15	De novo	g.93588232T>C	c.779T>C	p.(Phe260Ser)	Missense	LBD	0.75, 33
6	Unknown	g.93585389C>G	c.366C>G	p.(Cys122Trp)	Missense	DBD	0.935, 28.7
22	Unknown	g.93585377_93585380del	c.354_357del	p.(Leu118Phefs*33)	Frameshift	DBD	—, —
5	De novo	g.93585025T>C	c.2T>C	p.(Met1Thr)	Missense	TIC	0.53, 22.5
25	De novo	g.93585280G>T	c.257G>T	p.(Cys86Phe)	Missense	DBD	0.904, 32
7	De novo	g.93585376T>G	c.353T>G	p.(Leu118*)	Nonsense	DBD	‐, 37
16	De novo	g.93588221G>A	c.768G>A	p.(Trp256*)	Nonsense	LBD	—, —
4	Unknown	g.93585025_93585029del	c.2_6del	p.?	Start Loss	TIC	—, —
1	Unknown	g.93585340G>A	c.317G>A	p.(Cys106Tyr)	Missense	DBD	0.975, 32
4	Unknown	g.93585124_93585129delinsCCGGCGAGCAGCAGCA	c.101_106delinsCCGGCGAGCAGCAGCA	p.?	Frameshift	EW	—, —
27	Unknown	g.93585101_93585119del	c.78_96del	p.(Gln28Alafs*85)	Frameshift	EW	—, —
1	Unknown	g.93585343A>G	c.320A>G	p.(Lys107Arg)	Missense	DBD	0.909, 25.8
7	Unknown	g.93593687C>T	c.1117C>T	p.(Arg373*)	Nonsense	LBD	—, 39
4	Unknown	Whole gene deletion	—	—	Whole Gene Deletion	WGD	—, —
6	De novo	Whole gene deletion	—	—	Whole Gene Deletion	WGD	—, —
7	Unknown	g.93585412T>G	c.389T>G	p.(Ile130Ser)	Missense	DBD	0.949, 32
2	Unknown	g.93585309A>G	c.286A>G	p.(Lys96Glu)	Missense	DBD	0.801, 28.4
16	Unknown	g.93585486G>A	c.463G>A	p.(Ala155Thr)	Missense	EW	0.372, 35
12	De novo	g.93585383C>A	c.360C>A	p.(Tyr120*)	Nonsense	DBD	—, 36
5	De novo	Whole gene deletion	—	—	Whole Gene Deletion	WGD	—, —
1	De novo	g.93585083del	c.60del	p.Gly21Alafs*98	Frameshift	EW	—, —
7	De novo	Whole gene deletion	—	—	Whole Gene Deletion	WGD	—, —

Abbreviations: DBD = DNA‐binding domain; EW = elsewhere; F = female; LBD = ligand‐binding domain; M = male; TIC = translation‐initiation codon; WGD = whole gene deletion.

### 3.2 Phenotypes

**FIGURE 2:**
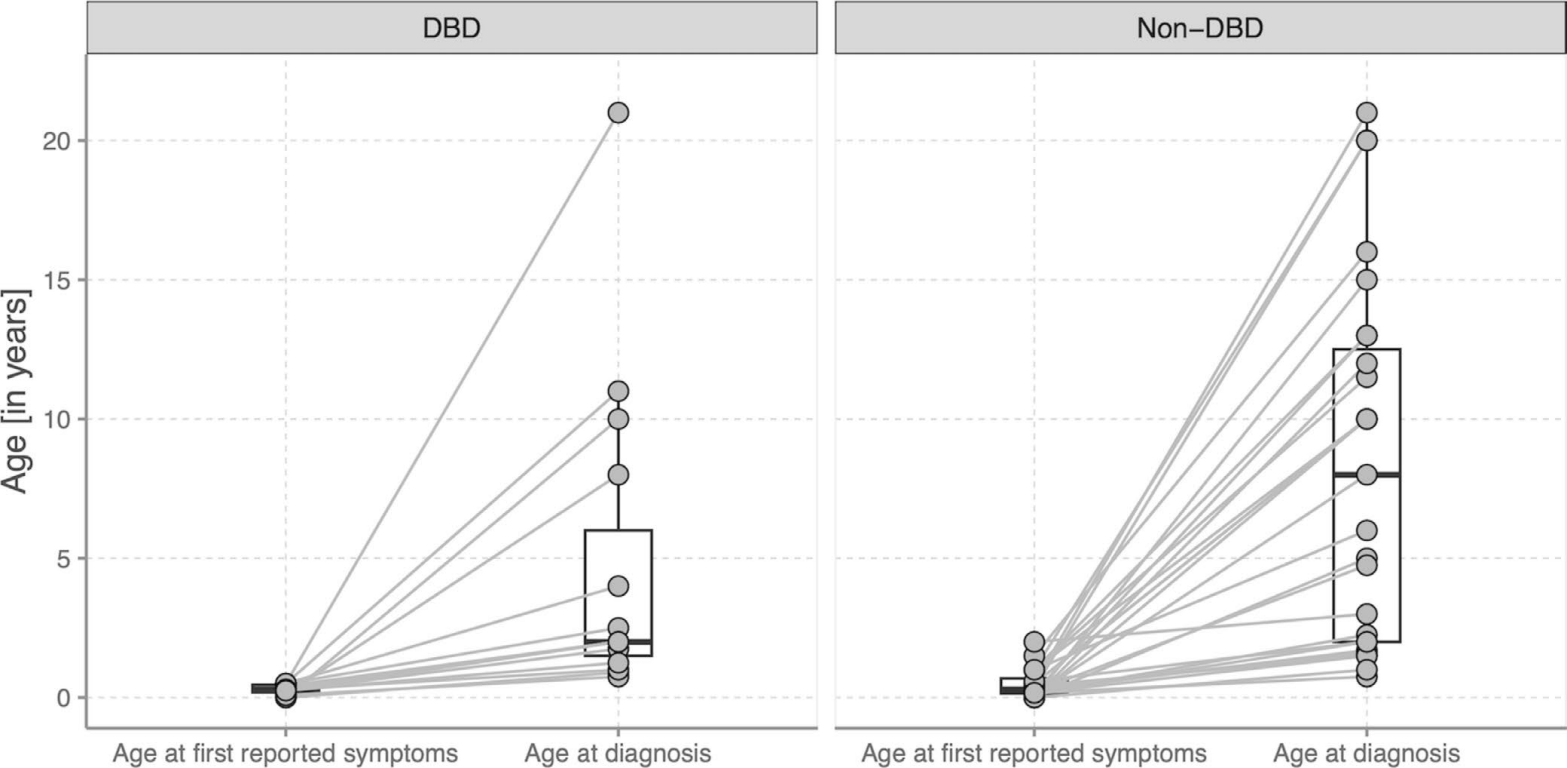
Age of first symptoms and age at BBSOAS diagnosis compared between DBD (*n* = 15) and non‐DBD (*n* = 28) group. Horizontal line represents median. The age at diagnosis differs statistically significantly between the two genotypic groups (mean 4.7 years vs. 8.9 years, *p* = 0.048).

The clinical phenotypes are shown in Table 3. In our cohort of 47 individuals, the most commonly reported features were developmental delay (speech delay in 95.7% (44/46), motor delay in 87.2% (41/47)), hypotonia (84.8%, 39/46), optic nerve atrophy (83.3%, 30/36), nystagmus (82.2%, 37/45), and strabismus (77.3%, 34/44). First symptoms were noticed on average at 4.9 months (SD 5.1). In most cases, the BBSOAS diagnosis was made many years after the onset of symptoms, with a mean age at diagnosis of 7.9 years (SD 7.0). The mean age at diagnosis differed statistically significantly between the two genotypic groups (DBD vs. non‐DBD, *p* = 0.048). As shown in Figure 2, in patients with variants in the DBD, first symptoms are reported on average around the 4th month of life (mean 0.29 years, SD ± 0.177, *n* = 15), while the BBSOAS diagnosis was made on average at around 4.7 years of age (mean 4.68 years, SD ± 5.63, *n* = 15). In patients with variants outside the DBD, the first symptoms are reported on average at around the 6th month of life (mean 0.47 years, SD ± 0.5, *n* = 28), while the diagnosis is made on average at around 8.9 years of age (mean 8.86 years, SD ± 7.1, *n* = 28).

#### 3.2.1 Developmental Milestones

Based on the milestones reported by the families, motor delay was present in 87.2% (41/47) and speech delay in 95.7% (44/46) of individuals. Six individuals did not show motor delay, two individuals reached their language milestones on time. No one reached the milestones of both domains, motor and speech, on time. Accordingly, all patients were developmentally delayed, in either motor or language or both domains. Individuals of the present study were able to sit on average at 13.5 months (SD 7.0), walk at 29.4 months (SD 21.3) and speak their first words (other than “mama” or “dada”) at 31 months (SD 20.4). Three individuals are not able to walk independently (at 4, 6, and 25 years of age), 9 individuals (9/45, 20%) are non‐verbal, with the oldest being 25 years of age. Language regression was reported in 30.6% (11/36).

All of the individuals aged ≥ 6 years old required special education (24/24, 100%), 57.1% (20/35) attended mainstream classes but required classroom modifications or additional resources.

#### 3.2.2 Neurology

Muscular hypotonia was reported as one of the most common features across the present cohort with 84.8% (39/46). Swallowing difficulties were reported in 40.9% (18/44), with a mean age of onset at 3.7 months (SD 4.6). Seizures were reported in 34.9% (15/43), with a mean age of onset at 5.2 years (SD 5.7). The latest onset of seizures was reported at 16 years of age. Febrile seizures occurred in 10.3% (4/39), the oldest being 3 years old. A subset of patients (19.5%, 8/41) had infantile spasms, predominantly individuals with variants in the DBD. Spasticity was reported in 5.6% (2/36), both individuals with variants in the non‐DBD group (one with a WGD, one with a variant elsewhere in the gene).

#### 3.2.3 Ophthalmology

Optic atrophy remains a key feature with 83.3% (30/36), although it is not the only ophthalmological symptom present. In the present cohort, the second most prevalent visual feature is nystagmus, followed by strabismus, visual field defects, optic nerve hypoplasia, and CVI. Refractive errors were also reported, with hyperopia being the predominant one.

These ophthalmological features were typically diagnosed during childhood. For example, CVI was diagnosed at a mean age of 4.5 years (SD 3.6) and optic atrophy at a mean age of 5.7 years (SD 5.8). The oldest patient diagnosed with optic atrophy was 22 years old.

#### 3.2.4 Behavior

Autistic features were present in 63.2% (24/38); 13 individuals had formal testing (ADOS, ADI‐R), of which 10 individuals met the criteria for formal ASD diagnosis.

Attention deficit was reported in 48.5% (16/33), hyperactivity in 32.4% (11/34), whereas 22.5% (9/40) were diagnosed with ADHD. Obsessive‐compulsive behavior (OCB) was reported in 35.3% (12/34). One individual had depression; no one in the present cohort reported bipolar disorder or schizophrenia.

#### 3.2.5 Other Features

Brain MRI was performed in 28 individuals, 16 of whom showed thin corpus callosum (57.1%, 16/28). Twenty‐nine individuals had an audiologic evaluation, 9 of them had a hearing deficit (31%, 9/29) but only two individuals required hearing aids. Subjective hearing problems were reported by 14.3% (6/42).

Congenital anomalies were reported by 23.9% of individuals (11/46), including bicuspid aortic valve, hydronephrosis, and vesicoureteral reflux. Coloboma was described in two patients; one had a coloboma of the iris, choroid, and optic disc, and one patient reported a unilateral chorioretinal‐ and iris‐coloboma.

### 3.3 Course of the Disorder

**FIGURE 3:**
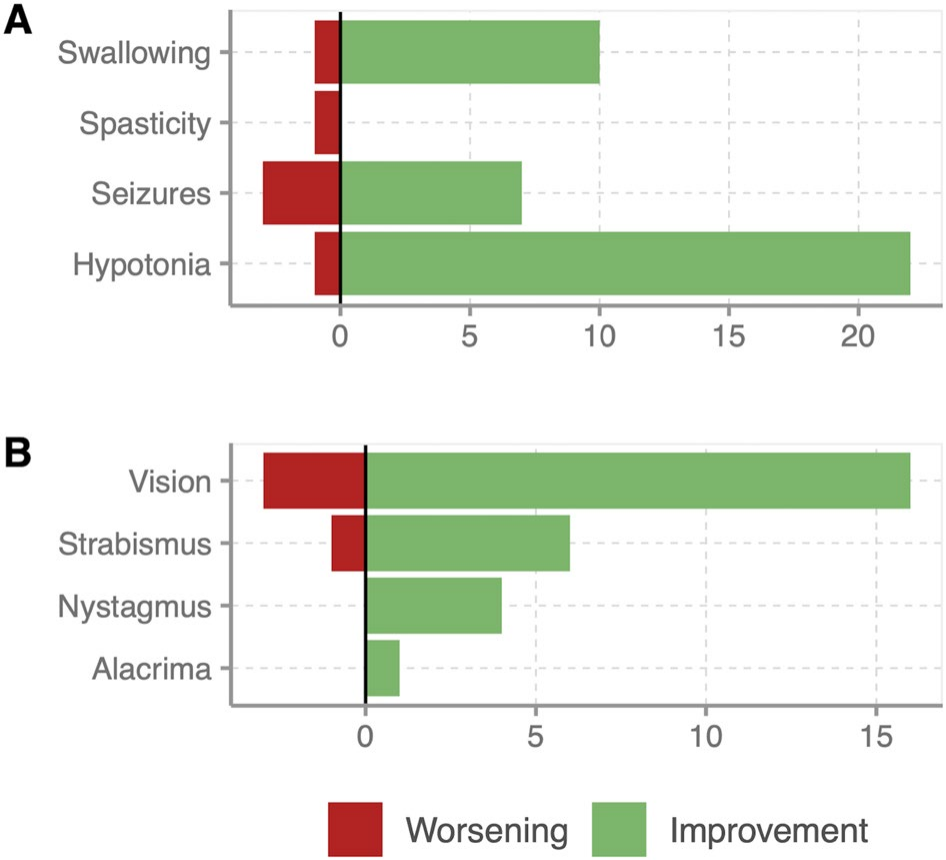
Graphical representation of reported improvement and worsening across all genotypic groups. (A) Selected neurological features. (B) Selected ophthalmological features.

To investigate the course of symptoms, we asked whether the feature had improved, worsened, or remained unchanged. The results are shown in Table 2 and Figure 3. Overall, more improvements were reported than deteriorations.

**TABLE 2:** Reported improvement and worsening of selected features across all genotypic groups.

Feature	Present, n	Improved, *n* (%)	Lower; upper 95% CI, %	Worsened, *n* (%)	Lower; upper 95% CI, %	Unknown, *n*
Hypotonia	39	22 (56.4)	39.6; 72.2	1 (2.6)	0.1; 13.5	16
Seizures	15	7 (46.7)	21.3; 73.4	3 (20.0)	4.3; 48.1	5
Spasticity	2	0	0.0; 84.2	1 (50.0)	1.3; 98.7	1
Swallowing	18	10 (55.6)	30.8; 78.5	1 (5.6)	0.1; 27.3	7
Alacrima	14	1 (7.1)	0.2; 33.9	0	0.0; 23.2	13
Nystagmus	37	4 (10.8)	3.0; 25.4	0	0.0; 9.5	33
Strabismus	34	6 (17.6)	6.8; 34.5	1 (2.9)	0.0; 15.3	27
Vision	34	16 (47.0)	29.8; 64.9	3 (8.8)	1.9; 23.7	15

*Note*: Percentage of improvement and worsening refer to the present data points. The label “unknown” means that the information about the course of that specific symptom is missing.

As shown in Table 2, improvement of hypotonia was reported by 22 probands, whereas hypotonia worsened in only one patient (hypotonia *n* = 39). Seizures improved in seven individuals and worsened in three (seizures *n* = 15). One individual reported a worsening of spasticity (spasticity *n* = 2). Another one reported a worsening of swallowing issues, whereas 10 patients described improvement in swallowing (swallowing problems *n* = 18). Improvement in vision was observed by 16 patients; 3 described a deterioration of vision (vision impairment *n* = 34). There was no worsening of alacrima and nystagmus; improvement was described in one case for alacrima (alacrima *n* = 14) and in four others for nystagmus (nystagmus *n* = 37). Strabismus improved in 6 patients and worsened in one (strabismus *n* = 34). The percentage of improvements and deteriorations was calculated in relation to the total number of patients with the respective symptom.

### 3.4 Genotype–Phenotype Correlations

**FIGURE 4:**
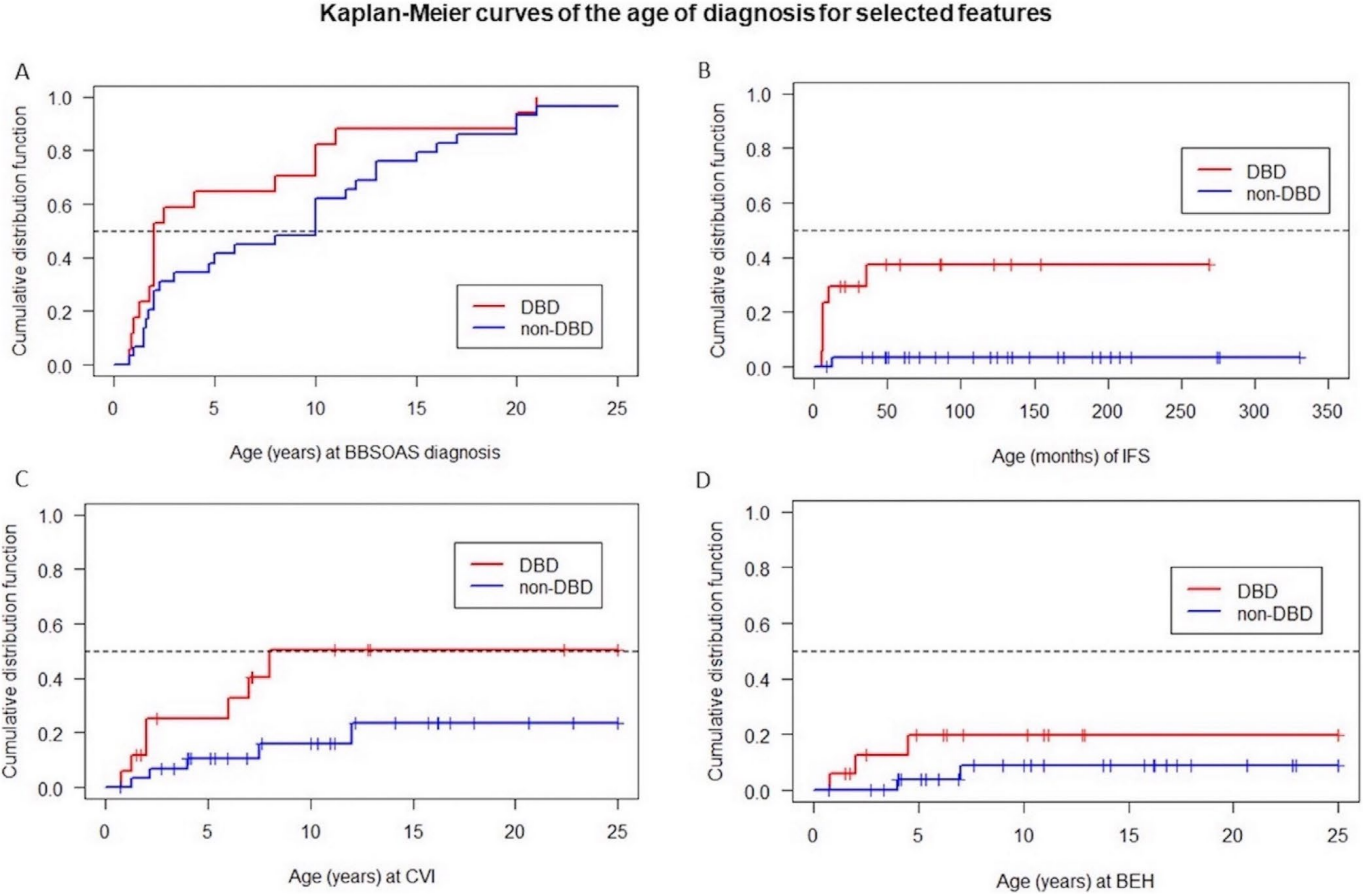
Dotted lines indicate the median line *y* = 0.5, vertical ticks mark censored observations. (A) 17 DBD and 29 non‐DBD patients, median age of BBSOAS diagnosis is 2 yrs. (95% CI: 1.4 yrs‐2.4 yrs) for DBD and 10 yrs. (95% CI: 5.9–14.1 yrs) for non‐DBD, Mann–Whitney *U*‐test *p* = 0.103. (B) 17 DBD and 30 non‐DBD patients, logrank‐test *p* = 0.002. (C) 17 DBD and 30 non‐DBD patients, logrank‐test *p* = 0.045. (D) 17 DBD and 30 non‐DBD patients, logrank‐test *p* = 0.184. For B, C, and D the median ages at infantile spasms, cerebral visual impairment, and behavioral problems as well as confidence intervals in DBD and non‐DBD groups cannot be calculated, since more than 50% did not show the respective feature and are censored observations. BEH = behavioral problems; CVI = cerebral visual impairment; IFS = infantile spasms.

To assess genotype–phenotype correlations, we conducted two different methodologies of data analysis. One was based on symptom frequency (Table 3), and the other was a time‐to‐event analysis (Figure 4). For the latter, we used the age at which the feature had occurred and, for the patients without the feature, the age at the end of the observation period (censored observations). We compared the time‐to‐event data between patients in the DBD group (*n* = 17) versus the non‐DBD (*n* = 30) group. The comparison between the three genotypic groups (DBD vs. TIC/WGD vs. LBD) is shown in Table S2.

**TABLE 3:** Clinical features overall and compared between DBD and non‐DBD group by Chi‐square test or Fisher's exact test in case of sparse data.

Phenotype	DBD	Non‐DBD	Overall	*p*
	(*n* = 17)	(*n* = 30)	(*n* = 47)	(two‐sided)
Development				
Motor delay	17/17 (100%)	24/30 (80%)	41/47 (87.2%)	0.074
Speech delay	16/16 (100%)	28/30 (93.3%)	44/46 (95.7%)	0.536
Non‐verbal	8/16 (50%)	1/29 (3.4%)	9/45 (20%)	**0.0004**
Neurology				
Seizures	6/15 (40%)	9/28 (32.1%)	15/43 (34.9%)	0.606
Infantile spasms	7/15 (46.7%)	1/26 (3.8%)	8/41 (19.5%)	**0.002**
Swallowing issues	6/15 (40%)	12/29 (41.4%)	18/44 (40.9%)	1
Hypotonia	14/16 (87.5%)	25/30 (83.3%)	39/46 (84.8%)	1
Ophthalmology				
CVI	8/10 (80%)	7/16 (43.8%)	15/26 (57.7%)	0.109
Optic atrophy	13/15 (86.7%)	17/21 (81%)	30/36 (83.3%)	1
Optic hypoplasia	6/11 (54.5%)	11/17 (64.7%)	17/28 (60.7%)	0.701
Strabismus	13/15 (86.7%)	21/29 (72.4%)	34/44 (77.3%)	0.452
Nystagmus	13/16 (81.3%)	24/29 (82.8%)	37/45 (82.2%)	1
Alacrima	5/13 (38.5%)	9/22 (40.9%)	14/35 (40%)	1
Audiology				
Hearing deficit in audiologic evaluation	3/11 (27.3%)	6/18 (33.3%)	9/29 (31.0%)	1
Hearing aids	0/13 (0%)	2/26 (7.7%)	2/39 (5.1%)	0.544
Behavior				
Autistic features	8/13 (61.5%)	16/25 (64.0%)	24/38 (63.2%)	1
Meets diagnostic criteria for ASD (formal testing)	5/6 (83.3%)	5/7 (71.4%)	10/13 (76.9%)	1
ADHD	1/15 (6.7%)	8/25 (32%)	9/40 (22.5%)	0.117
Other				
Mainstream school with resources	5/12 (41.7%)	15/23 (65.2%)	20/35 (57.1%)	0.282
Thin corpus callosum (brain MRI)	8/10 (80.0%)	8/18 (44.4%)	16/28 (57.1%)	0.114
Feeding difficulties (infancy)	10/16 (62.5%)	15/29 (51.7%)	25/45 (55.6%)	0.544
Eating difficulties (≥ 11 y)	2/5 (40%)	2/12 (16.7%)	4/17 (23.5%)	0.538
Good longterm memory (≥ 3 y)	8/10 (80%)	24/25 (96%)	32/35 (91.4%)	0.190
High pain tolerance (≥ 3 y)	13/14 (92.9%)	24/28 (85.7%)	37/42 (88.1%)	0.650
Touch sensitivity	10/13 (76.9%)	15/28 (53.6%)	25/41 (61%)	0.187
Sleeping difficulties	8/16 (50%)	13/29 (44.8%)	21/45 (46.7%)	0.765
Love of music	16/16 (100%)	26/30 (86.7%)	42/46 (91.3%)	0.282

*Note*: Significant values (*p* < 0.05) are shown in bold. Confidence intervals are shown in the supplement (Table S1).

Table 3 shows the number of occurrences of clinical features overall and compared between the DBD and the non‐DBD group. Although not statistically significant, children with variants in the DBD showed a greater prevalence in motor delay (100% vs. 80%, *p* = 0.074) and speech delay (100% vs. 93.3%, *p* = 0.536). As shown in Table 3, the DBD group had significantly more individuals that were non‐verbal (50% vs. 3.4%, *p* = 0.0004). Language regression was reported by 30.6% (11/36), with a higher prevalence in the DBD group (36% vs. 28%).

Regarding seizure history, there were significantly more children with variants in the DBD group that had infantile spasms (46.7% vs. 3.8%, *p* = 0.002). Two individuals were described as having spasticity, both in the non‐DBD group.

As mentioned above, 16/28 showed thinning of the corpus callosum on brain MRI. The prevalence of this feature was higher in the DBD group than in the non‐DBD group (80% vs. 44.4%).

The prevalence of optic atrophy/hypoplasia was relatively balanced between the two groups (86.7% vs. 81% for atrophy and 54.5% vs. 64.7% for hypoplasia).

While all children aged ≥ 6 years required special education, there were more children in the non‐DBD group who attended mainstream school with additional classroom modifications and resources (15/23, 65.2% in the non‐DBD group vs. 5/12, 41.7% in the DBD group).

As shown in Figure 4A, 50% of children in the DBD group were diagnosed before the age of 2 years, whereas in the non‐DBD group, 50% of individuals received their BBSOAS diagnosis before the age of 10 years, reflecting the later age of diagnosis in the non‐DBD group. The youngest age at diagnosis was 0.75 years; the oldest age at diagnosis was 25.4 years (SD 7.0). Figure 4B shows that infantile spasms occur predominantly in the DBD group and cluster in the first few months of life. In total, there were eight individuals with infantile spasms, seven of whom had variants in the DBD. The age of onset of infantile spasms is known for 7 children: 6/7 occurred within the first 12 months of life; one family reported onset at 36 months. As shown in Figure 4C, the youngest age at CVI diagnosis was 0.75 years, and the oldest age at CVI diagnosis was 12 years (SD 3.6).

## 4 Discussion

BBSOAS, a rare neurodevelopmental disorder caused by pathogenic variants in *NR2F1*, is characterized by DD/ID, visual impairment, hypotonia, epilepsy, behavioral anomalies such as autistic traits, and other manifestations [10]. To date, 92 individuals with BBSOAS have been described in the literature, showing a diverse genotypic and phenotypic spectrum [10]. Overall, the two most common features across these 92 individuals were DD and ID, with prevalences of 88.04% (DD) and 86.95% (ID) [10]. Since it was first described in 2014, much has been learned about the phenotype of BBSOAS. Initially described as a disorder causing intellectual disability with optic atrophy [2], the clinical spectrum of the disorder has steadily expanded. The vast majority of individuals published to date are children, although a few adults are known. One person has been reported at the age of 35 years [2]. There are indications that *NR2F1*‐associated optic neuropathy leading to visual impairment is not progressive [3]. However, little is known about the natural course of the disease. Here, we present 47 individuals with variants in *NR2F1*, with a special focus on whether and to what extent the symptoms of BBSOAS change over time.

Our study confirms characteristic clinical features of BBSOAS, such as DD, hypotonia, optic atrophy, nystagmus, strabismus, autistic features, and thinning of the corpus callosum on brain MRI. Consistent with previous descriptions, the dominant symptom in our study is DD, affecting all genotypic groups. Similar to Rech et al. [1], our BBSOAS children were able to sit on average 8 months later, walk 11 months later, speak their first words 17 months later, and combine words 15 months later than neurotypical children. The six individuals who did not show motor delay had variants outside the DBD. There were only two individuals who reached their speech milestones on time, one with a variant within the LBD and one within the TIC.

Previous publications showed the most severe clinical phenotype in patients that carry a variant in the DBD [1, 4]. Pathogenic variants in the DBD are expected to cause a dominant negative effect, while whole gene deletions and variants in the TIC are expected to result in haploinsufficiency [4, 10]. Consistent with previously reported findings [1], in our cohort, individuals with variants in the DBD showed higher prevalences of severe clinical features, such as infantile spasms (46.7% vs. 3.8%, *p* = 0.002) or being non‐verbal (50% vs. 3.4%, *p* = 0.0004). The age at diagnosis differs statistically significantly between the two genotypic groups (mean 4.7 years vs. 8.9 years, *p* = 0.048) complementing the statement of a more severe phenotype in the DBD group leading to an earlier diagnosis.

BBSOAS is known to be associated with different epileptiform pathologies, including epilepsy, infantile spasms, and febrile seizures [10]. Previous data reported that the prevalence of seizures is significantly greater in patients with variants in the DBD [1]. In our cohort, the prevalence of seizures is only slightly higher in the DBD group (40.0% vs. 32.1%), not reaching statistical significance with the exception of infantile spasms. Optic atrophy remains a key feature of BBSOAS with 83.3%. Optic neuropathies (atrophy and hypoplasia) reached high prevalences in both genotypic groups, showing no significant differences between these two, which is in line with previous data [1].

As a congenital disease, it can be assumed that most BBSOAS manifestations are already present at birth. However, it is not uncommon for patients to take years to receive their diagnosis. In our cohort, it was shown that individuals in the DBD group tend to get their diagnosis earlier than patients with other variants. In the DBD group, 50% of children were diagnosed before the age of 2 years, whereas in the non‐DBD group, 50% received their BBSOAS diagnosis before the age of 10 years, suggesting a milder phenotype in the non‐DBD group could lead to a diagnostic delay.

Until now, there has been limited understanding about the natural course of the disease. A recent study evaluating the *NR2F1*‐associated visual impairment could not find any evidence for progression but suggested the optic neuropathy to be congenital and stable [3].

Overall, the families reported more improvement than worsening. Although there are some cases of worsening, such as strabismus or hypotonia, the disease does not appear to be fundamentally progressive. Most symptoms show a trend towards improvement over time.

However, language regression presents a unique challenge. We assume that parents evaluate their children's language development by comparing to same‐aged peers. This dynamic comparison may introduce biases. On the one hand, an actual loss of previously acquired skills could have been observed. On the other hand, what may appear to be a regression could, in part, be a relative lag behind peers, potentially explaining why 30% of families reported having experienced language regression. Also, it should be noted that the prevalence of language regression among children with autism spectrum disorder is reported to be 20%–40% [12, 13].

Some of the improvements could be explained by various supportive therapies or compensational mechanisms. It is likely that the high rate of improvement in seizures may be due to medical treatment, but could also be related to the maturation of the central nervous system (CNS). Improvements in muscular hypotonia and swallowing might be attributed to physical and occupational therapy, but also due to CNS maturation. Furthermore, children with visual impairments may learn to compensate for their deficits to a certain extent.

At the present time, we cannot definitively answer whether this is the typical course of the disease. We are also unable to comment on whether and to what extent supportive treatment or medical intervention may influence the course of the disease, as this was not included in our questionnaire. As this would provide valuable information on possible interventions, this should be investigated further in subsequent studies.

The large number of unknowns in the “Course of disease” section in our study may be due to the technical method of the questionnaire itself. However, it could be assumed that no major changes were perceived, as otherwise we might have been able to capture these data points. We consider this plausible, although prospective follow‐up studies would be required to validate this.

To complement the information provided by the families, we asked them to send us medical documents. However, this study is based on information provided by the parents; standardized medical examinations of the patients are missing, and parental information may be subject to bias. Nevertheless, a medical study based on parental questionnaires enables a broad collection of valuable data with less effort—especially in studies of rare diseases with patients from all over the world—and provides important insights from the people who know the patients and their symptoms best.

As of now, there is no cure for BBSOAS, but the current data suggest that the disease is stable and non‐progressive. However, continued analyses, especially long‐term studies on the course of the features of BBSOAS, are necessary to further elucidate this. Our findings underscore the crucial role of early and sustained supportive therapy consisting of multidisciplinary specialists, highlighting the importance of thorough genetic testing for patients exhibiting related symptoms to ensure early diagnosis and timely intervention.

## Author Contributions

Conceptualization: C.P.S, I.V., and P.C. Methodology: I.V., P.C., and C.F. Investigation and data curation: C.F., I.V., H.B., and P.C. Writing – original draft preparation: I.V. and C.F. Writing – review and editing: C.P.S., C.F., H.B., and I.V. Visualization: H.B., C.F., and I.V. Supervising: C.P.S.

## Conflicts of Interest

The authors declare no conflicts of interest.

## Peer Review

The peer review history for this article is available at https://www.webofscience.com/api/gateway/wos/peer‐review/10.1111/cge.14731.

## Supporting information


**Table S1.** Confidence intervals of clinical features overall and compared between DBD and non‐DBD group.

**Table S2.** Clinical features overall and compared between the three genotypic groups (DBD = DNA‐binding domain, TIC = Translation‐initiation codon, WGD = Whole Gene Deletion, LBD = ligand‐binding domain, EW = elsewhere).

## Data Availability

The de‐identified data that support the findings of this study are available from the corresponding author upon reasonable request.
