# Supplementary material for: The Natural Course of Bosch‐Boonstra‐Schaaf Optic Atrophy Syndrome
Source: Clin Genet. 2025 Feb 19;108(2):168–78. doi: 10.1111/cge.14731 (PMC12215215; doi:10.1111/cge.14731)
Supplement: Supplementary file 1 — Table S1. Confidence intervals of clinical features overall and compared between DBD and non‐DBD group. [file CGE-108-168-s002.docx]

| **Feature** | **Genotypic group** | **k** | **n** | **rate** | **95-CI** |
| --- | --- | --- | --- | --- | --- |
| Motor delay | DBD | 17 | 17 | 1.000 | [0.8049; 1] |
|  | non-DBD | 24 | 30 | 0.800 | [0.6143; 0.9229] |
|  | total | 41 | 47 | 0.872 | [0.7426; 0.9517] |
| Speech delay | DBD | 16 | 16 | 1.000 | [0.7941; 1] |
|  | non-DBD | 28 | 30 | 0.933 | [0.7793; 0.9918] |
|  | total | 44 | 46 | 0.957 | [0.8516; 0.9947] |
| Nonverbal | DBD | 8 | 16 | 0.500 | [0.2465; 0.7535] |
|  | non-DBD | 1 | 29 | 0.034 | [0.0009; 0.1776] |
|  | total | 9 | 45 | 0.200 | [0.0976; 0.3460] |
| Seizures | DBD | 6 | 15 | 0.400 | [0.1634; 0.6771] |
|  | non-DBD | 9 | 28 | 0.321 | [0.1588; 0.5235] |
|  | total | 15 | 43 | 0.349 | [0.2101; 0.5093] |
| Infantile spasms | DBD | 7 | 15 | 0.467 | [0.2127; 0.7341] |
|  | non-DBD | 1 | 26 | 0.038 | [0.0010; 0.1964] |
|  | total | 8 | 41 | 0.195 | [0.0882; 0.3487] |
| Swallowing issues | DBD | 6 | 15 | 0.400 | [0.1634; 0.6771] |
|  | non-DBD | 12 | 29 | 0.414 | [0.2352; 0.6106] |
|  | total | 18 | 44 | 0.409 | [0.2634; 0.5675] |
| Hypotonia | DBD | 14 | 16 | 0.875 | [0.6165; 0.9845] |
|  | non-DBD | 25 | 30 | 0.833 | [0.6528; 0.9436] |
|  | total | 39 | 46 | 0.848 | [0.7113; 0.9366] |
| CVI | DBD | 8 | 10 | 0.800 | [0.4439; 0.9748] |
|  | non-DBD | 7 | 16 | 0.438 | [0.1975; 0.7012] |
|  | total | 15 | 26 | 0.577 | [0.3692; 0.7665] |
| Optic atrophy | DBD | 13 | 15 | 0.867 | [0.5954; 0.9834] |
|  | non-DBD | 17 | 21 | 0.810 | [0.5809; 0.9455] |
|  | total | 30 | 36 | 0.833 | [0.6719; 0.9363] |
| Optic hypoplasia | DBD | 6 | 11 | 0.545 | [0.2338; 0.8325] |
|  | non-DBD | 11 | 17 | 0.647 | [0.3833; 0.8579] |
|  | total | 17 | 28 | 0.607 | [0.4058; 0.7850] |
| Strabismus | DBD | 13 | 15 | 0.867 | [0.5954; 0.9834] |
|  | non-DBD | 21 | 29 | 0.724 | [0.5276; 0.8727] |
|  | total | 34 | 44 | 0.773 | [0.6216; 0.8853] |
| Nystagmus | DBD | 13 | 16 | 0.813 | [0.5435; 0.9595] |
|  | non-DBD | 24 | 29 | 0.828 | [0.6423; 0.9415] |
|  | total | 37 | 45 | 0.822 | [0.6795; 0.9200] |
| Alacrima | DBD | 5 | 13 | 0.385 | [0.1386; 0.6842] |
|  | non-DBD | 9 | 22 | 0.409 | [0.2071; 0.6365] |
|  | total | 14 | 35 | 0.400 | [0.2387; 0.5789] |
| Hearing deficit in audiologic evaluation | DBD | 3 | 11 | 0.273 | [0.0602; 0.6097] |
|  | non-DBD | 6 | 18 | 0.333 | [0.1333; 0.5901] |
|  | total | 9 | 29 | 0.310 | [0.1528; 0.5083] |
| Hearing aids | DBD | 0 | 13 | 0.000 | [0; 0.2471] |
|  | non-DBD | 2 | 26 | 0.077 | [0.0095; 0.2513] |
|  | total | 2 | 39 | 0.051 | [0.0063; 0.1732] |
| Autistic features | DBD | 8 | 13 | 0.615 | [0.3158; 0. 8614] |
|  | non-DBD | 16 | 25 | 0.640 | [0.4252; 0.8203] |
|  | total | 24 | 38 | 0.632 | [0.4599; 0.7819] |
| Meets diagnostic criteria (ASD, formal testing) | DBD | 5 | 6 | 0.833 | [0.3588; 0.9958] |
|  | non-DBD | 5 | 7 | 0.714 | [0.2904; 0.9633] |
|  | total | 10 | 13 | 0.769 | [0.4619; 0.9496] |
| ADHD | DBD | 1 | 15 | 0.067 | [0.0017; 0.3195] |
|  | non-DBD | 8 | 25 | 0.320 | [0.1495; 0.5350] |
|  | total | 9 | 40 | 0.225 | [0.1084; 0.3845] |
| Mainstream school with resources | DBD | 5 | 12 | 0.417 | [0.1517; 0.7233] |
|  | non-DBD | 15 | 23 | 0.652 | [0.4273; 0.8362] |
|  | total | 20 | 35 | 0.571 | [0.3935; 0.7368] |
| Thin corpus callosum (brain MRI) | DBD | 8 | 10 | 0.800 | [0.4439; 0.9748] |
|  | non-DBD | 8 | 18 | 0.444 | [0.2153; 0.6924] |
|  | total | 16 | 28 | 0.517 | [0.3718; 0.7554] |
| Feeding difficulties (infancy) | DBD | 10 | 16 | 0.625 | [0.3543; 0.8480] |
|  | non-DBD | 15 | 29 | 0.517 | [0.3253; 0.7055] |
|  | total | 25 | 45 | 0.556 | [0.4000; 0.7036] |
| Eating difficulties  (≥ 11y) | DBD | 2 | 5 | 0.400 | [0.0527; 0.8534] |
|  | non-DBD | 2 | 12 | 0.167 | [0.0209; 0.4841] |
|  | total | 4 | 17 | 0.235 | [0.0681; 0.4990] |
| Good longterm memory  (≥ 3y) | DBD | 8 | 10 | 0.800 | [0.4439; 0.9748] |
|  | non-DBD | 24 | 25 | 0.960 | [0.7965; 0.9990] |
|  | total | 32 | 35 | 0.914 | [0.7694; 0.9820] |
| High pain tolerance  (≥ 3y) | DBD | 13 | 14 | 0.928 | [0.6613; 0.9982] |
|  | non-DBD | 24 | 28 | 0.857 | [0.6733; 0.9597] |
|  | total | 37 | 42 | 0.881 | [0.7437; 0.9602] |
| Touch sensitivity | DBD | 10 | 13 | 0.769 | [0.4619; 0.9496] |
|  | non-DBD | 15 | 28 | 0.536 | [0.3387; 0.7249] |
|  | total | 25 | 41 | 0.610 | [0.4450; 0.7580] |
| Sleep difficulties | DBD | 8 | 16 | 0.500 | [0.2465; 0.7535] |
|  | non-DBD | 13 | 29 | 0.448 | [0.2645; 0.6431] |
|  | total | 21 | 45 | 0.467 | [0.3166; 0.6213] |
| Love of music | DBD | 16 | 16 | 1.000 | [0.7941; 1] |
|  | non-DBD | 26 | 30 | 0.867 | [0.6928; 0.9624] |
|  | total | 42 | 46 | 0.913 | [0.7921; 0.9758] |

Table S1. Confidence intervals of clinical features overall and compared between DBD and non-DBD group.
