# Supplementary material for: The Natural Course of Bosch‐Boonstra‐Schaaf Optic Atrophy Syndrome
Source: Clin Genet. 2025 Feb 19;108(2):168–78. doi: 10.1111/cge.14731 (PMC12215215; doi:10.1111/cge.14731)
Supplement: Supplementary file 2 — Table S2. Clinical features overall and compared between the three genotypic groups (DBD = DNA‐binding domain, TIC = Translation‐initiation codon, WGD = Whole Gene Deletion, LBD = ligand‐binding domain, EW = elsewhere). [file CGE-108-168-s001.docx]

| **Phenotype** | **DBD**  **(n=17)** | **TIC, WGD**  **(n=12)** | **LDB, EW**  **(n=18)** | **Overall**  **(n=47)** | **p-value**  **(two-sided)** |
| --- | --- | --- | --- | --- | --- |
| **Development** | | | | | |
| Motor delay | 17/17 (100%) | 10/12 (83.3%) | 14/18 (77.8%) | 41/47 (87.2%) | 0.137 |
| Speech delay | 16/16 (100%) | 11/12 (91.7%) | 17/18 (94.4%) | 44/46 (95.7%) | 0.722 |
| Nonverbal | 8/16 (50%) | 1/12 (8.3%) | 0/17 (0%) | 9/45 (20%) | 0.0005 |
| **Neurology** | | | | | |
| Seizures | 6/15 (40%) | 5/11 (45.5%) | 4/17 (23.5%) | 15/43 (34.9%) | 0.432 |
| Infantile spasms | 7/15 (46.7%) | 1/11 (9.1%) | 0/15 (0%) | 8/41 (19.5%) | 0.003 |
| Swallowing issues | 6/15 (40%) | 5/12 (41.7%) | 7/17 (41.2%) | 18/44 (40.9%) | 1.000 |
| Hypotonia | 14/16 (87.5%) | 9/12 (75.0%) | 16/18 (88.9%) | 39/46 (84.8%) | 0.589 |
| **Ophthalmology** | | | | | |
| CVI | 8/10 (80%) | 4/8 (50.0%) | 3/8 (37.5%) | 15/26 (57.7%) | 0.213 |
| Optic atrophy | 13/15 (86.7%) | 7/9 (77.8%) | 10/12 (83.3%) | 30/36 (83.3%) | 0.861 |
| Optic hypoplasia | 6/11 (54.5%) | 8/10 (80.0%) | 3/7 (42.9%) | 17/28 (60.7%) | 0.340 |
| Strabismus | 13/15 (86.7%) | 8/12 (66.7%) | 13/17 (76.5%) | 34/44 (77.3%) | 0.487 |
| Nystagmus | 13/16 (81.3%) | 10/12 (83.3%) | 14/17 (82.4%) | 37/45 (82.2%) | 1.000 |
| Alacrima | 5/13 (38.5%) | 6/11 (54.5%) | 3/11 (27.3%) | 14/35 (40%) | 0.478 |
| **Audiology** | | | | | |
| Hearing deficit in audiologic evaluation | 3/11 (27.3%) | 3/9 (33.3%) | 3/9 (33.3%) | 9/29 (31.0%) | 1.000 |
| Hearing aids | 0/13 (0%) | 1/11 (9.1%) | 1/15 (6.7%) | 2/39 (5.1%) | 0.737 |
| **Behavior** | | | | | |
| Autistic features | 8/13 (61.5%) | 6/10 (60.0%) | 10/15 (66.7%) | 24/38 (63.2%) | 1.000 |
| Meets diagnostic criteria for ASD (formal testing) | 5/6 (83.3%) | 2/2 (100%) | 3/5 (60.0%) | 10/13 (76.9%) | 0.528 |
| ADHD | 1/15 (6.7%) | 2/9 (22.2%) | 6/16 (37.5%) | 9/40 (22.5%) | 0.131 |
| **Other** | | | | | |
| Mainstream school with resources | 5/12 (41.7%) | 5/9 (55.6%) | 10/14 (71.4%) | 20/35 (57.1%) | 0.358 |
| Thin corpus callosum (brain MRI) | 8/10 (80.0%) | 4/7 (57.1%) | 4/11 (36.4%) | 16/28 (57.1%) | 0.151 |
| Feeding difficulties (infancy) | 10/16 (62.5%) | 6/12 (50.0%) | 9/17 (52.9%) | 25/45 (55.6%) | 0.803 |
| Eating difficulties (≥ 11y) | 2/5 (40%) | 1/3 (33.3%) | 1/9 (11.1%) | 4/17 (23.5%) | 0.445 |
| Good longterm memory (≥ 3y) | 8/10 (80%) | 9/10 (90%) | 15/15 (100%) | 32/35 (91.4%) | 0.174 |
| High pain tolerance (≥ 3y) | 13/14 (92.9%) | 11/12 (91.7%) | 13/16 (81.2%) | 37/42 (88.1%) | 0.603 |
| Touch sensitivity | 10/13 (76.9%) | 6/10 (60.0%) | 9/18 (50.0%) | 25/41 (61%) | 0.338 |
| Sleep difficulties | 8/16 (50%) | 5/12 (41.7%) | 8/17 (47.1%) | 21/45 (46.7%) | 0.932 |
| Love of music | 16/16 (100%) | 11/12 (91.7%) | 15/18 (83.3%) | 42/46 (91.3%) | 0.228 |

Table S2. Clinical features overall and compared between the three genotypic groups (DBD = DNA-binding domain, TIC = Translation-initiation codon, WGD = Whole Gene Deletion, LBD = ligand-binding domain, EW = elsewhere).
